# GABA-mediated regulation of lipid metabolism: a conserved relation with diverse physiological significances

**DOI:** 10.3389/fphys.2026.1801052

**Published:** 2026-05-29

**Authors:** Samjhana Pradhan, Sanaly Nava, Kristina Hill, Javier Ochoa-Repáraz, Kavita Sharma

**Affiliations:** 1 Department of Chemistry, Idaho State University, Pocatello, ID, United States; 2 Department of Biomedical and Pharmaceutical Sciences, Idaho State University, Pocatello, ID, United States; 3 Department of Medical Laboratory Science, Idaho State University, Meridian, ID, United States; 4 Department of Biological Sciences, Boise State University, Boise, ID, United States

**Keywords:** adipogenesis, algae, anti-diabetic, biofuel, chilling injury, lipogenesis, lipolysis, stress tolerance

## Abstract

Gamma-aminobutyric acid (GABA) is a four-carbon non-proteinogenic amino acid that is primarily known for its role as an inhibitory neurotransmitter in mammals. It is synthesized from glutamate by the enzyme L-glutamic acid decarboxylase and metabolized via the GABA shunt pathways. GABA is known to influence various metabolic processes, and recent studies have shown its influence on lipid metabolism. This review explores the distinct relationship between GABA and lipid metabolism by highlighting their role in physiological processes in animals, plants, and microalgae. In animals, GABA acts as a metabolic regulator, particularly in the liver, where it mitigates diseases associated with fat accumulation, such as obesity, hyperlipidemia, and type 2 diabetes mellitus, by modulating adipogenesis and lipogenesis (fat synthesis and accumulation), lipolysis (fat breakdown), and thermogenesis and oxidation (energy expenditure). Conversely, in plants and algae, it plays a vital role in stress responses and developmental processes by suppressing lipid membrane degradation and by promoting lipid accumulation. We also delve into the metabolic pathways through which GABA interacts with lipid metabolism, including its connection to the tricarboxylic acid cycle and its involvement in the GABA shunt. This review underscores the multifaceted nature of GABA, revealing its critical contribution to lipid homeostasis, highlighting its relevance in metabolic disorders in animals, and stress responses in both plants and algae. Despite being a relatively underexplored area, the GABA-lipid relationship holds substantial significance due to its involvement in varied physiological processes, suggesting promising avenues for future therapeutic research and biotechnological applications.

## Graphical Abstract

**Figure:**
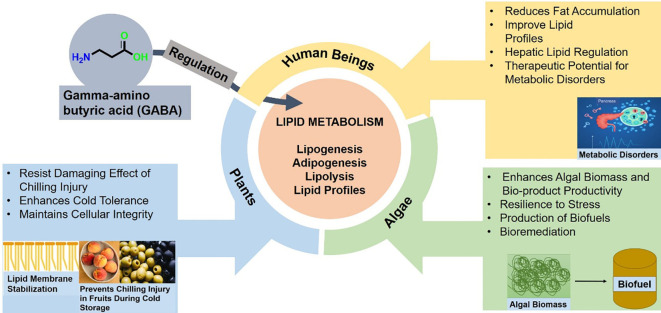

## 1 Introduction

Gamma-aminobutyric acid (GABA) is a ubiquitous four-carbon non-proteinogenic amino acid that serves as the primary inhibitory neurotransmitter in the central nervous system (CNS) (Chen and Sharma, 2025). Its fundamental role is to reduce neuronal excitability, essentially slowing down brain activity by blocking specific signals in the CNS (Deng et al., 2009). GABA is synthesized from the excitatory neurotransmitter glutamate through the action of the enzyme glutamic acid decarboxylase (GAD). Once released, GABA exerts its inhibitory effects through two distinct receptors; the ionotropic GABA_A_ and the metabotropic GABA_B_ (Dastgerdi et al., 2021). GABA_A_ receptors function as ligand-gated ion channels that facilitate the rapid influx of chloride ions (Cl^-^), directly hyperpolarizing the neuron. In contrast, GABA_B_ receptors uses G-protein-coupled signaling pathway to increase potassium (K^+^) conductance, allowing positive ions to exit the cell. Both processes drive the membrane potential toward a more negative state, resulting in shunting inhibition, thereby dampening overall neuronal excitability and stabilizing the neural circuit (Chen and Sharma, 2025). However, in animals, GABA’s influence extends far beyond the CNS; it functions as a critical signaling molecule in peripheral metabolic organs, including the liver (Franklin and Wollheim, 2004; Hwang et al., 2019), pancreas (Jin and Korol, 2023), and adipose tissues (Jin et al., 2024; Zhu et al., 2019). The correlation between GABA levels and the progression of metabolic diseases underscores its systemic relevance (Zhu et al., 2019; Su et al., 2024). In particular, the systemic importance of GABA in maintaining physiological balance is expanding due to its potential antioxidant (Wang et al., 2018; Luo et al., 2023), anti-apoptotic (Bhat et al., 2010; Bhandage et al., 2018), and anti-inflammatory properties (Khan et al., 2021; Li et al., 2021a).

The biological significance of GABA is not limited to animals; its presence across algae and plants suggests a fundamental role in all life forms. In prokaryotes, most notably GABA-producing bacteria such as *Lactobacillus brevis* and *Bifidobacterium adolescentis*, GABA synthesis is not a metabolic byproduct but a survival strategy, primarily mediated by the GAD system which consists of a decarboxylase enzyme (GadB) and a glutamate/GABA antiporter (GadC) (Ramesh et al., 2016; Arora et al., 2023). When bacteria encounter highly acidic environments, such as the human stomach or fermented food matrices, the GAD system functions as a critical metabolic buffer. During this process, decarboxylation of L-glutamate into GABA consumes an intracellular proton (H^+^), directly raising the internal pH. The GadC antiporter then exports GABA in exchange for a new glutamate molecule. This exchange helps maintain a proton motive force, ensuring cellular homeostasis under extreme stress (Guo et al., 2013). Beyond acid resistance, GABA provides significant metabolic plasticity. Certain bacteria utilize GABA as a primary carbon and nitrogen source through the GABA shunt, which converts GABA into succinate for entry into the tricarboxylic acid (TCA) cycle (Seifikalhor et al., 2022). This allows GABA-producing bacteria to thrive even in nutrient-limited environments. In the human gut microbiome, this ability of microbes to synthesize and metabolize GABA can potentially influence host health and physiology via the gut-brain axis (Carillo and Carra, 2025).

Similarly, in plants, GABA is a vital metabolite, governing growth, development, and defense mechanisms, against environmental stressors (Mazzucotelli et al., 2006; Purwana et al., 2014; Su et al., 2019; Seifikalhor et al., 2020). Under adverse conditions such as drought (Hagan et al., 2022; De Bie et al., 2023), salinity (Xu et al., 2022), cold (Xu et al., 2022), and heavy metal exposure (Begazo-Jimenez et al., 2025), GABA rapidly accumulates to enhance stress tolerance (Fahy et al., 2011). This accumulation facilitates the regulation of intracellular pH and ion transport while activating antioxidant systems to scavenge reactive oxygen species (ROS), thereby mitigating stress-induced damage (Fahy et al., 2011). Plant GABA synthesis occurs primarily via cytoplasmic GAD or through the GABA shunt and polyamine degradation pathways (Mazzucotelli et al., 2006). Crucially, its metabolism is deeply integrated with carbon and nitrogen balance, providing essential carbon skeletons and energy for various biosynthetic processes, including those related to lipids (Mazzucotelli et al., 2006; Seifikalhor et al., 2020). Through these diverse biological processes, GABA plays a comprehensive role in modulating the metabolism of glucose (Eyster, 2007; Garcia et al., 2023), insulin (Liu et al., 2022), polyamines (Köberlin et al., 2016), and lipids (Cooper, 2000; Evans and Hauton, 2016). This metabolic regulation is pivotal for maintaining energy balance, insulin sensitivity, fat storage, and cellular response to metabolic stress (Cooper, 2000; Eyster, 2007; Evans and Hauton, 2016; Köberlin et al., 2016; Liu et al., 2022; Garcia et al., 2023). Central to these metabolic networks are lipids, a broad class of functional organic molecules comprising glycerophospholipids, glycerolipids, sphingolipids, fatty acyls, and polyketides (Parhofer, 2015). Lipids are indispensable to biological systems, functioning as regulators of signal transduction (Santos and Preta, 2018) and modulators of cellular responses via receptors such as G-protein coupled receptors (Ischebeck et al., 2020), toll-like receptors, and cytokine receptors (Kong et al., 2019). Structurally, lipids, particularly phospholipids, maintain cell membrane integrity (Kugler et al., 2019), while triacylglycerols (TAGs) serve as major long-term energy reserves (Banaszak et al., 2024). Given these multifunctional roles, lipid homeostasis is vital. In animals, dysregulation of lipid metabolism is a precursor to disorders such as obesity, metabolic syndrome, and cardiovascular diseases (Sharma et al., 2021; Kim and Yoon, 2023). Similarly, in plants and algae, lipids are essential for energy storage, signaling, and structural integrity (Olsen and DeLorey, 2016). Hence, imbalances here can disrupt growth, development, and defense responses. Furthermore, lipids in algae represent a significant feedstock for biodiesel production (Fait et al., 2008) and a rich source of polyunsaturated fatty acids (PUFAs), which support human health by reducing inflammation (Sanders and Griffin, 2015; Saponaro et al., 2015).

Despite the established individual importance of GABA and lipids, the regulatory influence of GABA on lipid metabolism remains a complex and underexplored subject. While recent comprehensive reviews have detailed the general biosynthesis and broad physiological functions of GABA (Zhang et al., 2024), emerging evidence suggests that GABA may mediate changes in lipid metabolism across various biological forms. GABA can act as a signaling bridge between stress response and energy reserve, and potentially influence lipid composition and accumulation rates to ensure survival under stress and maintain metabolic health. Understanding this crosstalk holds profound significance, as it could unlock new strategies for understanding stress adaptation and developing novel therapeutic and biotechnological applications. Therefore, this review aims to explore the multifaceted mechanisms by which GABA influences lipid metabolism across animals, plants, and algae. By highlighting the physiological and biological significance of this relationship, we aim to provide fundamental insights into the potential of leveraging the GABA-lipid connection. Investigating this intricate connection will not only advance our understanding of metabolic regulation but also contribute to the development of interventions for human health and sustainable advancements in the bio-economy.

## 2 Metabolic cross-talk: the intersections of GABA and lipid metabolism

### 2.1 GABA metabolism and its link to central carbon pathways

**Figure 1:**
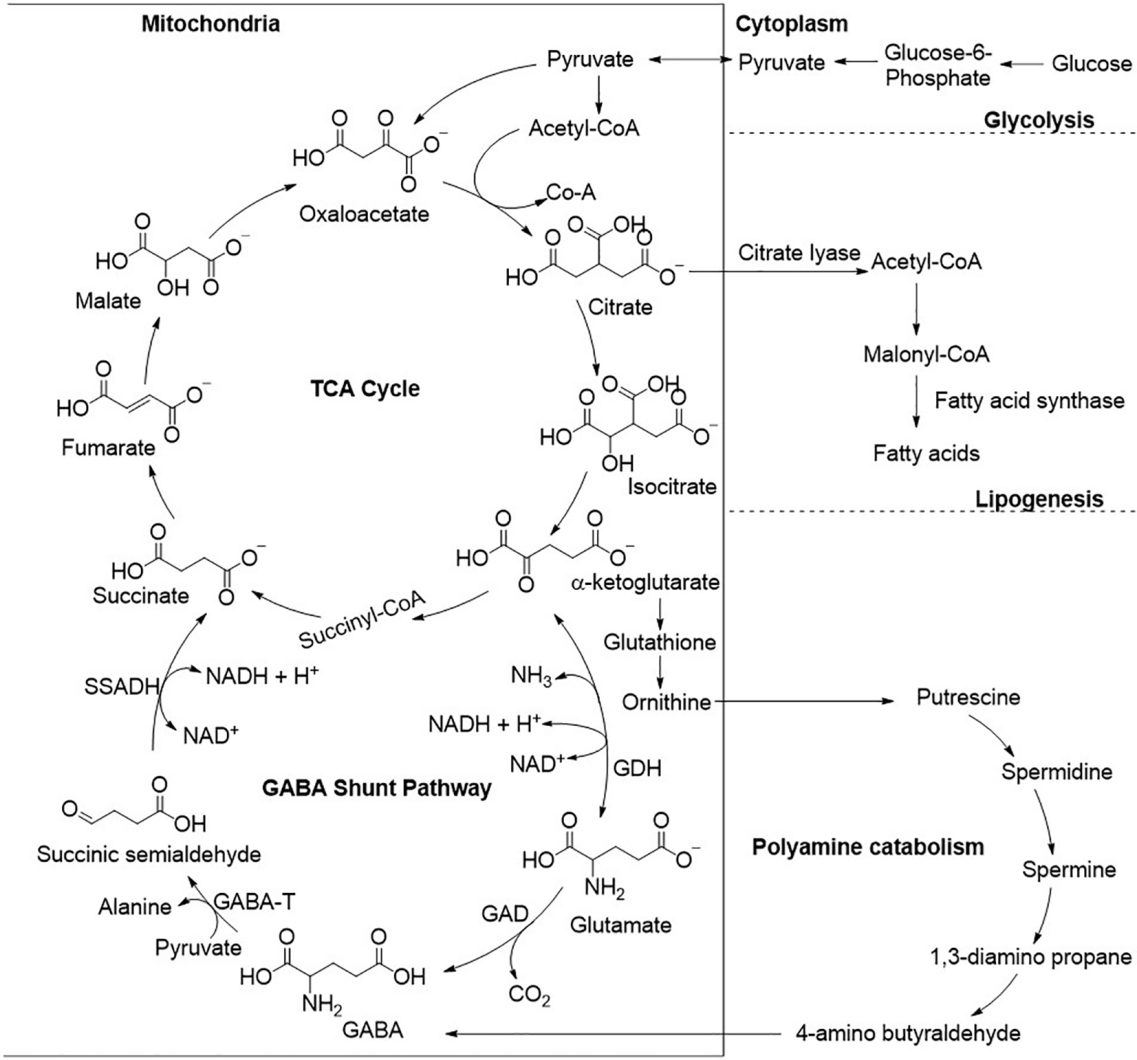
Integration of GABA shunt with the TCA cycle and lipid metabolism.

The metabolism of GABA is intrinsically linked to the central carbon and nitrogen balance, primarily through a three-step metabolic sequence known as the GABA shunt. GABA is synthesized from the excitatory amino acid glutamate via the irreversible decarboxylation catalyzed by GAD. Once synthesized, GABA is not simply an end product; it is fed back into the TCA cycle for energy generation through the GABA shunt. As shown in the GABA shunt pathway, **Figure 1**, GABA serves as a metabolic bridge rather than a mere end product. It is reintegrated into the TCA cycle through a two-step conversion. First, GABA is converted to succinic semialdehyde (SSA) by the enzyme GABA transaminase (GABA-T). SSA is then rapidly oxidized to succinate by succinic semialdehyde dehydrogenase (SSADH). Succinate is a key intermediate in the TCA cycle (Krebs cycle). By feeding succinate directly into the TCA cycle, the GABA shunt bypasses two key enzymatic steps of the TCA cycle (isocitrate dehydrogenase and α-ketoglutarate dehydrogenase) (De Biase and Pennacchietti, 2012; Yogeswara et al., 2020). This crucial metabolic rerouting, highlighted in the lower portion of **Figure 1**, demonstrates how the bypass directly affects the cell’s energy status and availability of carbon skeletons. Because the TCA cycle is the major source of precursors for biosynthesis, variations in the GABA shunt activity can subsequently impact the pool of metabolic intermediates, including those required for lipid synthesis.

### 2.2 Lipid metabolism: biosynthesis (lipogenesis) and breakdown (lipolysis)

Lipids are primarily supplied to the body through dietary intake, mobilization from stored adipose tissue, and *de novo* synthesis (lipogenesis). Lipogenesis, the process of synthesizing lipids, primarily occurs in the liver and adipose tissue when energy intake exceeds demand, often due to high carbohydrate or glucose consumption (Rai et al., 2025). The central molecule driving this process is acetyl-CoA, as shown in **Figure 1**. When excess glucose is metabolized, it generates a surplus of acetyl-CoA (Dover and Halpern, 1972). If this supply overwhelms the capacity of the TCA cycle to generate ATP, the excess acetyl-CoA is channeled toward the synthesis of various lipids in the cytosol via the citrate molecule. Specifically, acetyl-CoA is the foundational precursor for the synthesis of fatty acids (via fatty acid synthase), triglycerides (storage lipids), cholesterol, and steroid hormones (**Figure 1**). Conversely, when energy is needed, lipolysis occurs. This is the catabolic breakdown of stored triglycerides into glycerol and free fatty acids (FFAs). These FFAs are then oxidized through β-oxidation to produce acetyl-CoA, which subsequently enters the TCA cycle for ATP generation. The balance between lipogenesis and lipolysis is critical for maintaining lipid homeostasis and preventing metabolic disorders (Strandwitz et al., 2018). In this regard, the link between GABA and lipids becomes evident. GABA shunt acts upstream by feeding succinate directly into the TCA cycle, which in turn modulates the flow of intermediates and the resulting availability of acetyl-CoA for lipogenesis. Therefore, GABA levels can be seen as an indirect regulator of the carbon flux that determines whether a cell commits to energy production (TCA cycle) or energy storage (lipogenesis), thereby governing overall lipid profiles and cellular membrane integrity.

## 3 GABA and lipid metabolism

### 3.1 Animals

Lipid metabolism is a fundamental biological process critical for maintaining life, energy balance, cellular structure, and overall health in animals. However, abnormalities in lipid synthesis, storage, and catabolism are primary drivers of many chronic diseases, contributing to severe conditions such as obesity, insulin resistance, type 2 diabetes mellitus (T2DM), metabolic-associated fatty liver disease (MAFLD), and hepatic steatosis, cardiovascular diseases, and neurodegenerative disorders (Xu et al., 2024). Liver is the primary metabolic organ for lipid processing, where GABA signaling plays a crucial role in mitigating MAFLD and hepatic steatosis (Chen et al., 2022). This regulation is largely mediated through the expression of ionotropic GABA_A_ receptors on hepatocytes and resident Kupffer cells (Cui et al., 2025). Activation of hepatic GABA_A_ receptors facilitates chloride ion influx, which in Kupffer cells has been shown to suppress TLR4/NF-κB-mediated inflammatory signaling and reduce TNF-α production, thereby preventing the transition from simple steatosis to severe inflammatory states (Cui et al., 2025). Simultaneously, GABA triggers a signaling cascade—often involving the phosphorylation of AMP-activated protein kinase (AMPK)—that effectively shifts the liver from energy storage to energy expenditure (Lee et al., 2022). These receptor-mediated pathways alleviate steatosis by suppressing *de novo* lipogenesis through the downregulation of sterol regulatory element-binding protein 1c (SREBP-1c) and fatty acid synthase (FAS). Furthermore, GABA signaling enhances the expression of genes involved in β-oxidation, such as PPARα, while also regulating hepatic GABA-T-mediated flux, which has recently been identified as a critical marker of metabolic health in the liver (Geisler et al., 2021; Cui et al., 2025).

#### 3.1.1 Effects on lipid homeostasis

GABA has emerged as a potent metabolic regulator capable of improving lipid profiles and preventing detrimental fat accumulation, particularly in conditions associated with high-fat diets (HFD), obesity, and diabetes (Chen et al., 2016; Jiang et al., 2021; Zhang et al., 2022; Jin et al., 2024; Xiao et al., 2024; Han et al., 2025). Research utilizing pure GABA (Jin et al., 2024) and GABA-rich products, such as fermented yogurts and adzuki beans (Chen et al., 2016; Jiang et al., 2021; Zhang et al., 2022; Xiao et al., 2024; Han et al., 2025), consistently demonstrates strong anti-obesity and lipid-lowering capabilities. **Table 1** provides a comprehensive summary of these findings, detailing how supplementation of GABA and GABA-rich products modulate lipid homeostasis across animal models and cell lines. It specifically highlights improvement in serum lipid profiles and shift toward lipolysis, and suppression of adipogenesis and lipogenesis, demonstrating the therapeutic potential of GABA in maintaining metabolic diseases. In HFD-induced obese C57BL/6J mice, pure GABA administered orally (30 mg/kg/day), reduced body mass gain, white adipose tissue (WAT) mass, and adipocyte size, alongside improvements in dyslipidemia, which is characterized by a reduction in serum triglycerides (TGs), total cholesterol (TC), and LDL cholesterol. In this case, GABA regulated lipid metabolism by suppressing the expression of adipogenic and lipogenic proteins, such as peroxisome proliferator-activated receptor gamma (PPARγ), CCAAT/enhancer-binding protein alpha (C/EBPα), and FAS. Simultaneously, it also promoted energy expenditure through the upregulation of lipolytic enzymes (PKA (protein kinase A), adipose triglyceride lipase (ATGL), and thermogenic proteins like uncoupling protein 1 (UCP1) (Jin et al., 2024).

**Table 1:** Impact of GABA on lipid metabolism in animals.

Condition/Model	GABA products used	Impact on serum lipid profile	Impact on tissue and organ fat	Key metabolic observations	References
Streptozotocin-Induced Diabetic Mice	GABA-rich yogurt fermented by *Streptococcus salivarius* subsp. *thermophiles* fmb5	Increased HDL cholesterol; decreased total cholesterol and triglycerides.	Normalized hypertrophy of the liver and kidney; restored pancreas islet integrity.	Improved insulin secretion (35 % increase) and enhanced glucose tolerance.	Chen et al., 2016
3T3-L1 Adipocytes (*in vitro* fat cells)	*Lactobacillus brevis*-fermented GABA	NA (Cell study)	Inhibited overall lipid accumulation and reduced size/number of lipid droplets in adipocytes; Suppressed differentiation of preadipocytes into mature fat cells.	• Inhibited adipogenesis/lipogenesis by downregulating master fat formation proteins like PPARγ and FABP4, as well as lipogenic enzymes like FAS and lipin 1. • Promoted lipolysis by activating the PKA signaling pathway and upregulating lipolytic enzymes like ATGL and p-HSL. • Induced browning by upregulating thermogenic markers UCP1, CPT1 and PPARα for promoting energy expenditure.	Han et al., 2025
Oleic Acid-induced HepG2 Cells (*in vitro* fatty liver model)	GABA-rich Okara supernatant	NA (Cell study)	Reduced intracellular total cholesterol and triglycerides accumulation; Improved oxidative stress by reducing ROS and malondialdehyde.	• Inhibited lipogenesis by downregulating proteins such as SREPB-1, FAS, ACC1, and SCD1. • Activated phosphorylated AMPK and PPARα to accelerate fatty acid oxidation. • Reduced oxidative stress by activating the Keap1/Nrf2/HO-1 signaling pathway.	Xiao et al., 2024
Type 2 Diabetic Mice	GABA-rich sprouted adzuki beans	Decreased triglycerides and total cholesterol.	Prevented weight gain; Alleviated pathological changes in liver and cecal tissues; Reduced liver injury markers.	Gut microbiota regulation by reducing the Firmicutes/Bacteroidetes ratio (associated with obesity) and increasing beneficial bacteria, *Akkermansia*.	Zhang et al., 2022
Type 2 Diabetic Mice	GABA-rich germinated adzuki beans	Decreased triglycerides and total cholesterol.	Recovered liver and kidney functional indicators such as aspartate aminotransferase, alanine aminotransferase, and urea.	• Improved glycerophospholipid and sphingolipid metabolism. • Regulated tryptophan metabolism. • Downregulated L-serine and L-histidine levels to lower blood glucose.	Jiang et al., 2021
High-Fat Diet-Induced Obese Mice	Pure GABA (Oral administration)	Decreased triglycerides, total cholesterol and LDL cholesterol.	Decreased body mass gain and fat mass; reduced size of subcutaneous and visceral white adipose tissue; suppressed hepatic steatosis.	• Inhibited adipogenesis/lipogenesis by downregulating PPARγ, C/EBPα, FAS, and SERBP1. • Promoted lipolysis and browning by activating PKA pathway and upregulating UCP1.	Jin et al., 2024

Similar molecular mechanisms were confirmed using GABA-rich fermented products. In an *in vitro* model using 3T3-L1 adipocytes, the administration of *Lactobacillus brevis*-fermented GABA (LB-GABA, 100 ug/mL) reduced the number and size of lipid droplets and inhibited the differentiation of pre-adipocytes into mature fat cells. Molecular analysis showed that LB-GABA downregulated key fat synthesis markers like PPARγ, fatty acid-binding protein 4 (FABP4), and lipin 1, while activating the PKA signaling pathway to enhance lipolysis and upregulating thermogenic markers such as UCP1 and carnitine palmitoyltransferase 1 (CPT1) (Han et al., 2025). Furthermore, using a streptozotocin (STZ)-induced diabetic mouse (SIDM) model, GABA-rich yogurt fermented by *Streptococcus salivarius subsp. thermophiles* fmb5 (administered orally, 4 g/L) not only improved hyperglycemia and insulin secretion but also significantly ameliorated lipid metabolism disorders. It also decreased serum TC and TGs concentrations, coupled with an increase in beneficial HDL cholesterol. Similarly, the treatment normalized organ hypertrophy in the liver and kidney, and restored pancreatic islet function, suggesting a systemic protective effect against metabolic complications (Chen et al., 2016).

Likewise, in a T2DM model established via HFD and STZ injection in C57BL/6J mice, GABA, delivered through GABA-rich sprouted adzuki beans, achieved effective weight control and a significant reduction in serum TG (32.9%) and TC (48.8%) along with improved liver function markers. Serum metabolomic analysis revealed that this intervention regulated key metabolic pathways, including glycerophospholipid and sphingolipid metabolism while gut microbiota analysis showed a favorable shift in the microbial community, characterized by a reduced Firmicutes/Bacteroidetes ratio and increased abundance of the beneficial bacterium *Akkermansia* (Zhang et al., 2022). Building on these observations, direct intervention with GABA-producing *Lactobacillus brevis* strains (DPC6108 and DSM32386) has been shown to attenuate metabolic dysfunction through the elevation of luminal GABA levels. In diet-induced obese mice, these GABA-secreting microbes significantly increased GABA concentrations in the small intestine, which was associated with a reduction in mesenteric adipose tissue accumulation and improved plasma cholesterol clearance, demonstrating that endogenous microbial GABA production can modulate systemic lipid homeostasis and mitigate physiological abnormalities associated with metabolic syndrome (Patterson et al., 2019). Similarly, when treating OA-induced HepG2 cells (a human liver cell line induced with oleic acid to simulate fatty liver) with GABA-rich supernatant from fermented okara (FOS-G), it reduced intracellular lipid accumulation, specifically TC and TGs, while improving oxidative stress markers by reducing ROS and malondialdehyde levels. In this case, FOS-G inhibited hepatic lipogenesis by downregulating proteins such as SREBP-1, FAS, and stearoyl-CoA desaturase 1, and promoted fatty acid oxidation by activating the AMPK signaling pathway and upregulating PPARα expression (Xiao et al., 2024).

The systemic and local molecular improvements brought by GABA and GABA-rich products is detailed in **Table 2**, which categorizes the specific genes and proteins modulated by GABA across three distinct metabolic axes. GABA inhibits adipogenesis and lipogenesis by downregulating the master transcription factors PPARγ and C/EBPα, which prevents the differentiation of precursor cells into mature cells, a fat-storing adipocytes. This suppression is reflected at the enzymatic level, where a reduction in FAS, ACC1, and SCD1 prevents the synthesis of new fatty acids and triglycerides. The outcome of these down regulations, along with decreased levels of the carrier protein FABP4 and the esterification enzyme DGAT1, is a significant impact on the body’s capacity to expand fat tissue and store excess energy. Simultaneously, GABA accelerates the mobilization of existing energy by promoting lipolysis. By increasing the phosphorylation (activation) of pPKA, GABA initiates metabolic degradation, and as a result upregulates key lipases, including ATGL, pHSL, and MGL. This shift activates fat breakdown, resulting in rapid hydrolysis of stored triglycerides into free fatty acids and glycerol. This ensures that the energy previously stored in white adipose tissue is released back into the system for utilization. Finally, GABA ensures that these released lipids are permanently released through enhanced thermogenesis and fatty acid oxidation. This is achieved by inducing the browning of white fat cells, a process driven by the upregulation of mitochondrial markers such as UCP1, PGC1α, and PRDM16. By uncoupling mitochondrial respiration, UCP1 allows the cell to dissipate energy as heat rather than storing it as ATP. To fuel this thermogenic demand, GABA upregulates PPARα and CPT1, which facilitate the transport and burning of fatty acids within mitochondria. This entire process is overseen by the activation of pAMPK, a metabolic sensor that maintains energy balance by switching the cell from a fat-storing state to a fat-burning state.

**Table 2:** Specific gene and protein expressions modulated by GABA and GABA-rich products.

Molecular mechanism	Gene/Protein	Function	Effect of GABA	References
Inhibition of adipogenesis and lipogenesis: GABA reduces obesity by suppressing the expression of transcription factors and enzymes responsible for creating new fat cells and synthesizing triglycerides.	PPARγ (Peroxisome proliferator-activated receptor gamma)	A master regulator of adipogenesis which drives the formation of mature fat cells.	Downregulated (reduced expression inhibits fat cell formation)	Jin et al., 2024; Han et al., 2025
	C/EBPα (CCAAT/enhancer-binding protein alpha)	Works with PPARγ to promote adipocyte differentiation.		
	FABP4 (Fatty acid-binding protein 4)	A carrier protein for fatty acids; a marker of mature fat cells involved in lipid accumulation.		
	SREBP-1 (Sterol regulatory element-binding protein 1)	A transcription factor that activates genes required for fatty acid and triglyceride synthesis.		
	FAS (Fatty acid synthase)	An enzyme that catalyzes the synthesis of fatty acids (fat building blocks).		
	ACC1 (Acetyl-CoA carboxylase 1)	A rate-limiting enzyme in fatty acid synthesis.		
	SCD1 (Stearoyl-CoA desaturase 1)	Converts saturated fatty acids into monounsaturated fatty acids (fat storage).		
	DGAT1 (Diacylglycerol O-acyltransferase 1)	A key enzyme crucial for adipogenesis (and lipid metabolism, catalyzing the final step in creating triglycerides from diacylglycerol and fatty acyl-CoA, storing energy and influencing fat accumulation in tissues like adipose tissue.		
Promotion of lipolysis (Fat breakdown): GABA stimulates the breakdown of stored triglycerides into free fatty acids and glycerol by activating the PKA signaling pathway.	p-PKA (Phosphorylated protein kinase A)	The master switch that activates lipolysis.	Upregulated (increased phosphorylation activates the pathway)	
	ATGL (Adipocyte triglyceride lipase)	It initiates the breakdown of triglycerides.		
	p-HSL (Phosphorylated hormone-sensitive lipase)	Breaks down diglycerides into monoglycerides; activated by PKA.		
	MGL (Monoacylglycerol lipase)	The final enzyme that breaks down monoglycerides into free fatty acids and glycerol.		
Enhance thermogenesis and oxidation (energy expenditure): GABA encourages the body to waste energy as heat rather than storing it, primarily by inducing the browning of white fat cells.	UCP1 (Uncoupling protein 1)	The key thermogenic protein that uncouples respiration from ATP production, releasing energy as heat.	Upregulated (indicates browning of white adipose tissue)	Jin et al., 2024; Han et al., 2025; Xiao et al., 2024
	PPARα (Peroxisome proliferator-activated receptor alpha)	Promotes the uptake and oxidation (burning) of fatty acids in the liver and adipose tissue.		
	CPT1 (Carnitine palmitoyltransferase 1)	Transports fatty acids into mitochondria to be burned (oxidized).		
	PGC1α (PPARγ coactivator 1-alpha)	A master regulator of mitochondrial biogenesis and thermogenesis.		
	PRDM16 (positive regulatory domain zinc finger region protein 16)	A transcriptional coregulator that drives the development function of heat-producing brown and beige adipocytes, which burn energy to produce heat.		
	p-AMPK (Phosphorylated AMPK)	An energy sensor that switches on catabolic pathways (burning fat) and switches off anabolic pathways (storing fat).		

#### 3.1.2 Mechanism of GABA-induced lipid metabolism

The impact of GABA on serum lipid profiles and hepatic condition is due to its capacity to regulate the metabolic balance, shifting the body from energy storage toward energy expenditure. This metabolic shift is primarily achieved by suppressing adipogenesis and lipogenesis, the processes responsible for fat accumulation (Jiang et al., 2021; Zhang et al., 2022; Jin et al., 2024; Xiao et al., 2024; Han et al., 2025). Studies involving both pure GABA and GABA-rich extracts have demonstrated a significant reduction in lipid accumulation within cellular models (Xiao et al., 2024; Han et al., 2025) and a corresponding decrease in adipose tissue mass in high-fat diet animal models (Chen et al., 2016; Jiang et al., 2021; Zhang et al., 2022; Jin et al., 2024). These anti-obesity effects are driven by the downregulation of master transcriptional regulators, such as PPARγ and FABP4 (Negri et al., 2022), alongside the inhibition of critical enzymes required for the synthesis of fatty acids and triglyceride synthesis, including FAS, acetyl-CoA carboxylase, and diacylglycerol acyltransferase1 (DGAT1) (Yen et al., 2008). Furthermore, the regulation of FABP4 by GABA modulates the proteasomal degradation of PPARγ, preventing pathological adipocyte expansion (Garin-Shkolnik et al., 2014). The downregulation of these lipogenic pathways and transcription factors is largely coordinated by the activation of master metabolic switches. A central component of this regulatory network is the GABA-mediated activation of the AMPK signaling pathway (Wang et al., 2021; Xiao et al., 2024), which may occur through three primary molecular routes. The Ca^2+^/CaMKKβ pathway, key upstream kinase, is one of the primary route that can directly phosphorylate and activate AMPK at the Thr^172^ residue (Hawley et al., 2005). While GABA is an inhibitory neurotransmitter, its interaction with both GABA_A_ and GABA_B_ receptors have been reported to increase intracellular calcium (Ca^2+^) levels across diverse cell types, including non-neuronal and specialized neuronal cell types, via depolarization-induced influx and metabotropic-induced internal release (Dave and Bordey, 2009; Constantin et al., 2010; Negri et al., 2022; Bostel et al., 2025). This rise in calcium acts as a secondary messenger that activates calmodulin-dependent protein kinase kinase β (CaMKKβ) (Marcelo et al., 2016) to activate the AMPK.

GABA supplementation further sustains AMPK activity by upregulating the expression and activity of sirtuin 1 (SIRT1) (Prud’homme et al., 2014), an NAD^+^ dependent deacetylase. SIRT1 facilitates the deacetylation and activation of liver kinase B1 (LKB1), which serves as a primary master kinase for AMPK (Lizcano et al., 2004). However, unlike the CaMKKβ activation route, LKB1-mediated activation of AMPK operates independently of calcium ion channels (Shaw et al., 2004). GABA also exerts influence through systemic endocrine signaling. Supplementation has been shown to enhance the secretion of adiponectin from adipose tissue (Weerawatanakorn et al., 2023). Adiponectin acts as an autocrine and endocrine messenger (Dadson et al., 2011) that binds to AdipoR1 receptors, triggering a secondary wave of AMPK activation and phosphorylation (Weerawatanakorn et al., 2023). This pathway is particularly relevant for improving insulin sensitivity and suppressing pro-inflammatory cytokine production, reinforcing the anti-obesogenic environment. Once activated, AMPK functions as a cellular energy sensor that effectively blocks the entire lipogenic cascade by suppressing SREBP-1 and PPARγ which are required for the synthesis and storage of triacylglycerols (Kersten, 2001).

Beyond inhibiting fat storage, GABA facilitates the mobilization and utilization of existing fat reserves by lipolysis where stored triglycerides are broken down into glycerol and free fatty acids for use as fuel. This process is mediated through the activation of the PKA signaling pathway, which triggers the phosphorylation and activation of essential lipolytic enzymes such as hormone-sensitive lipase (HSL) (Greenberg et al., 2001) and ATGL (Miyoshi et al., 2007). Once these free fatty acids are released, GABA further promotes their oxidation by increasing the expression of PPARα and CPT1. Because CPT1 serves as the rate-limiting route for transporting long-chain fatty acids in the mitochondria, its upregulation ensures that these lipids are efficiently burned via β-oxidation rather than being re-esterified for storage (Jin et al., 2024; Han et al., 2025). Complementing these pathways, GABA increases overall energy expenditure by inducing the browning of energy-storing WAT into energy-burning beige adipocytes. This transformation is a pivotal strategy in combating obesity, as it converts tissues designed for storage into metabolic processors. This effect is similarly driven by the PKA signaling pathway, which stimulates the expression of UCP1. As the defining feature of brown and beige fat, UCP1 uncouples mitochondrial respiration from the production of ATP, allowing the energy derived from lipid oxidation to be dissipated as heat through thermogenesis (Jin et al., 2024; Han et al., 2025). By simultaneously repressing fat synthesis and activating lipolytic and thermogenic pathways, GABA provides a comprehensive mechanism to mitigate fat accumulation and its associated metabolic disorders.

#### 3.1.3 Physiological outcomes

GABA regulates the metabolic disorder in animals, leading to a healthier phenotype across multiple organ systems. This intervention results in a systemic shift away from fat storage and toward energy utilization. The most prominent outcome is a significant reduction in adiposity and body mass gain in obese models, which is pathologically confirmed by a decrease in the overall size and number of adipocytes (fat cells) in WAT (Jin et al., 2024; Han et al., 2025). Crucially, GABA promotes thermogenesis by driving the browning of WAT, effectively increasing the animal’s energy expenditure and contributing to fat mass reduction. At the systemic level, GABA corrects the hallmark of metabolic disease by successfully ameliorating dyslipidemia (Chen et al., 2016; Jin et al., 2024; Han et al., 2025). This is evidenced by a consistent lowering of serum TG and TC while simultaneously achieving an increase in beneficial HDL-C. This improved lipid profile is intrinsically linked to glucose homeostasis, manifesting as significantly decreased fasting blood glucose and improved insulin sensitivity. Furthermore, GABA provides significant organ protection against lipid toxicity. It suppresses hepatic lipogenesis, thereby preventing or alleviating hepatic steatosis (fatty liver disease) (Jin et al., 2024; Xiao et al., 2024). This protective effect is supported by a reduction in serum markers of liver damage. Furthermore, GABA-rich products normalize the pathological enlargement (hypertrophy) of the liver and kidney (Chen et al., 2016), and aid in restoring the integrity of pancreatic islets, suggesting a broad systemic benefit in delaying the progression of diabetes-related complications.

### 3.2 Plants

**Figure 2:**
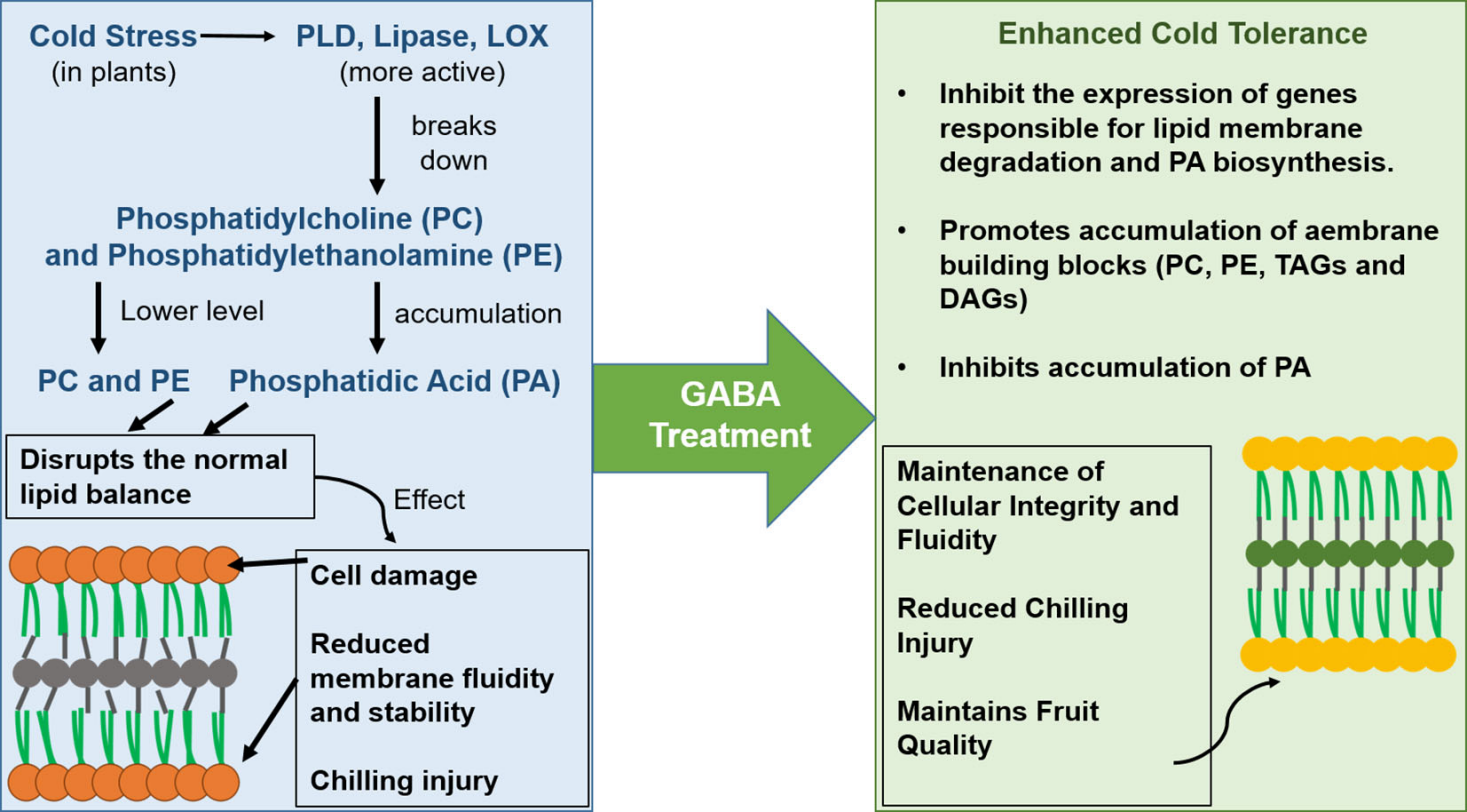
Schematic representation of GABA-mediated enhancement of cold tolerance in plants.

Lipids store energy, maintain the integrity and fluidity of cell membranes, and provide protective barriers against environmental stress in plants (Kim, 2020). Phospholipids, such as phosphatidylcholine (PC), phosphatidylethanolamine (PE), and phosphatidic acid (PA), are structural lipids that maintain membrane permeability and fluidity; storage lipids, like diacylglycerol (DAG) and TAGs, are part of the lipid membrane for maintaining cellular integrity (Van et al., 2008). Under environmental stress, such as low temperatures (chilling), this lipid membrane balance is severely disrupted. As shown in **Figure 2**, at low temperatures, plants often experience rapid accumulation of PA, which can damage cell membranes (Wu et al., 2022). The membranes undergo a phase transition from a flexible liquid-crystalline state to a rigid gel structure. This phase separation often triggers the degradation of structural lipids, leading to the accumulation of toxic lipid intermediates and loss of cellular compartmentalization (Marangoni et al., 1996), resulting in chilling injury. Therefore, maintaining a dynamic balance between these different lipid classes is critical for the plant’s ability to defend against cold stress. **Figure 2** further shows how GABA supplementation mitigates this damage. By modulating the lipidome, GABA prevents toxic accumulation of PA and promotes synthesis of stabilizing phospholipids like PC. This intervention preserves membrane fluidity and prevents the loss of cellular compartmentalization, thereby preventing chilling-induced injury and maintaining cell integrity.

#### 3.2.1 Effects of GABA on lipid homeostasis

GABA is a natural signaling molecule in plants that has been confirmed to effectively improve tolerance to stressors like cold. A key part of this protective function involves GABA’s ability to regulate membrane lipid metabolism, which has been identified as a novel mechanism for enhancing stress tolerance (Wang et al., 2025). Application of GABA enhances cold tolerance and preserves fruit quality during postharvest storage. In cold-sensitive species like Chinese olives (Chen et al., 2024) and peaches (Song et al., 2022), chilling injury typically triggers a cascade of membrane degradation, yet exogenous GABA treatment effectively counters this process by maintaining the structural and functional integrity of the cellular barrier. The efficacy of this treatment is concentration-specific. In the study of Chinese olives stored at 2^°^C, for instance, screening of multiple concentrations (0.5, 1.0, 1.5, and 2.0 mM) identified 1.0 mM GABA application as the optimal dose, with higher concentrations showing a reduced protective effect (Chen et al., 2024). Similarly, a 5.0 mM concentration of GABA was most effective for Hujing peaches (Song et al., 2022). While low to moderate doses alleviate stress, excessively high concentrations of GABA can be detrimental. In plant species such as *Arabidopsis thaliana*, supra-optimal GABA levels, often exceeding 10 mM, have been associated with growth inhibition and suppression of root elongation (Renault et al., 2011). This growth inhibition is largely attributed to defects in cell elongation and downregulation of genes essential for maintaining cell wall integrity, including expansins and arabinogalactan proteins, as well as disruptions in carbon-nitrogen balance and hormonal crosstalk involving abscisic acid, ethylene, and auxin (Renault et al., 2011; Podlešáková et al., 2019). Furthermore, excessive GABA accumulation can trigger the buildup of ROS and metabolic intermediates like succinic semialdehyde, which may inadvertently exacerbate oxidative stress and lead to physiological damage, including chlorosis and programmed cell death (Podlešáková et al., 2019). Hence, by applying specific doses, fruits can better withstand low-temperature stress, resulting in significantly lower cell membrane permeability and reduced accumulation of malondialdehyde (MDA), a primary indicator of oxidative damage.

The impact of GABA treatment on plant lipid profiles are detailed in **Table 3**. It summarizes how GABA treatment modulates plant lipids to alleviate chilling injury during storage. By promoting key structural phospholipids like PC and PE, GABA maintains membrane stability and cellular integrity. This effect is reinforced by increased fatty acid unsaturation, which prevents the membrane from becoming rigid under cold stress. Furthermore, GABA inhibits degradation pathways by suppressing PLD and LOX activity. This prevents the premature breakdown of lipid bilayer and toxic accumulation of PA. During prolonged cold exposure, GABA also enhances TAG accumulation that serves as energy source. Ultimately, the functional significance of these changes is reflected in reduced MDA levels and lower membrane permeability, which shows the relation between lipid regulation and mitigation of chilling injury.

**Table 3:** Impact of GABA on lipid metabolism in plants.

Lipid/Enzyme	Properties	Impact of GABA treatment	Effect/Significance	References
Phosphatidylcholine and Phosphatidylethanolamine	Membrane structural lipids	Maintained or increased their levels	Preserves the integrity and fluidity of the cell membrane, which is critical for resisting cold-induced damage.	Chen et al., 2024; Song et al., 2022; Khaliq et al., 2023; Aghdam et al., 2016; Ngaffo et al., 2021
Phosphatidic acid (PA)	Membrane signaling lipids	Reduced or inhibited accumulation	PA is associated with stress signaling and degradation pathways. Reducing its accumulation helps stabilize the membrane in the early stages of cold stress.	
Phospholipid degradation/PA Biosynthesis Pathways	Lipid metabolism regulation	Inhibition	Prevents the breakdown of structural phospholipids and the synthesis of stress-signaling PA, directly protecting the cell membrane from destabilization.	Chen et al., 2024; Song et al., 2022
Diacylglycerol and Triacylglycerol	Storage/Protective lipids	Enhanced accumulation (in later stages)	Provides a protective buffer and potential energy reserve against prolonged low-temperature stress, contributing to enhanced cold tolerance.	
Fatty Acids	Fatty acid saturation	Increased unsaturation	Maintains membrane fluidity in cold conditions.	Chen et al., 2024; Aghdam et al., 2016; Ngaffo et al., 2021
Lipid enzymes		Inhibits PLD and LOX	Stops enzymatic degradation of the membrane.	Chen et al., 2024; Song et al., 2022; Khaliq et al., 2023; Aghdam et al., 2016
Cell membrane permeability / Malondialdehyde (MDA) Content	Physiological outcome	Significantly reduced	Directly demonstrates the functional success of lipid regulation, resulting in lower cell damage (MDA) and greater membrane stability, which alleviates chilling injury.	Song et al., 2022

#### 3.2.2 Mechanism of GABA-induced lipid metabolism

During exposure to low temperatures, plant cell membranes often undergo accelerated de-esterification, where structural phospholipids are hydrolyzed into pro-oxidative and signaling molecules that exacerbate cellular damage. GABA acts as a metabolic regulator, preventing this degradative process to preserve the membrane integrity. It does this by suppressing the activity of key hydrolytic enzymes, including PLD, lipase, and LOX (Aghdam et al., 2016; Song et al., 2022; Khaliq et al., 2023; Chen et al., 2024), which catalyze the breakdown of essential membrane phospholipids. Likewise, by downregulating the expression of genes such as *PpPLDα4*, *PpPLDγ1*, and *PpLPAAT2*, GABA prevents the rapid breakdown of structural phospholipids like PC, PE, and phosphatidylinositol (PI) (Song et al., 2022). This inhibition is crucial because it limits the production of PA, a signaling lipid when present in excess, destabilizes the membrane bilayer and promotes a transition from a fluid state to a rigid, leaky gel state. Furthermore, GABA attenuates PA biosynthesis through the downregulation of diacylglycerol kinase (PpDGK) and lysophosphatidic acid acyltransferase (PpLPAAT), ensuring that the membrane remains in a stable environment for cellular processes (Song et al., 2022).

In addition to preventing degradation, GABA actively modulates the fatty acid composition to preserve membrane fluidity under cold stress. The treatment maintains high levels of unsaturated fatty acids, such as oleic (C18:1), linoleic (C18:2), and linolenic (C18:3) acids while simultaneously restricting the accumulation of saturated fatty acids like palmitic (C16:0) and stearic (C18:0) acids (Aghdam et al., 2016; Ngaffo et al., 2021; Song et al., 2022; Khaliq et al., 2023). This results in a higher index of unsaturated fatty acids and a favorable ratio of unsaturated to saturated lipids (Aghdam et al., 2016; Song et al., 2022), which prevents the membrane from becoming brittle and crystallizing in the cold. Beyond these structural adjustments, in fruits like peaches, GABA also triggers a unique remodeling process where membrane-derived lipids are sequestered into neutral storage forms, such as TAGs and DAGs. This conversion is largely facilitated by the upregulation of the phospholipid diacylglycerol acyltransferase gene (*PpPDAT*) (Song et al., 2022). This pathway effectively serves as a metabolic buffer, allowing the plant to temporarily store lipid building blocks in an inert form. During recovery or prolonged cold exposure, these stored lipids can be recycled to repair and restabilize the membrane, essential for mitigating the internal browning associated with postharvest chilling injury. The involvement of GABA in lipid regulation can be further supported through intricate crosstalk with key phytohormones, such as abscisic acid (ABA). GABA treatment often triggers an increase in endogenous ABA levels and modulates the expression of core signaling genes, including the ABA receptor PYL4 (pyrabactin resistance 1-like 4), the protein phosphatases such as abscisic acid-insensitive 1 (ABI1) and ABI2, and the protein kinase OST1 (open stomata 1, a member of the SnRK2 (sucrose non-fermenting 1-related protein kinase 2 family) (Liu et al., 2021). This activation of the ABA signaling cascade facilitates the binding of ABA to the PYR/PYL/RCAR (regulatory components of ABA receptors) receptors, which then interact and inhibit PP2C phosphatases. This inhibition releases the SnRK2/OST1 kinases to phosphorylate downstream transcription factors such as ABF3 (ABRE-binding factor 3) and ABI5. These activated transcription factors directly regulate lipid metabolism by binding to ABA-responsive elements (ABRE) located within the promoter regions of fatty acid desaturase (FAD) genes, such as EgFAD2 (Shi et al., 2021). Furthermore, FAD2 expression is strongly co-expressed with a network of transcription factors, including WRI1 (Wrinkled 1), bZIP (basic leucine zipper), MYB (myeloblastosis), and WRKY, as well as other fatty acid biosynthesis genes like FATA (fatty acyl-ACP thioesterase A), FATB, LACS (long-chain acyl-CoA synthetase), and SAD (stearoyl-ACP desaturase) (Shi et al., 2021). Hence, through the regulation of the GABA-ABA-FAD pathway, plants can enhance the expression of desaturases and other biosynthesis enzymes, leading to the accumulation of unsaturated fatty acids, such as linoleic acid, thereby reinforcing membrane structural integrity and mitigating stress-induced damage.

#### 3.2.3 Physiological outcomes

GABA improves the physiological quality and shelf life of cold-stored fruits, specifically Chinese olives and peaches, by mitigating chilling injury. The symptoms of chilling injury, such as internal browning, flesh mealiness in peaches, and browning/discoloration in Chinese olives, are alleviated due to the regulation of molecular and metabolic functions exerted by GABA. These lipid adjustments preserve cellular compartmentalization, which is the primary defense against enzymatic browning. When the membrane bilayer remains intact and fluid, the cell can effectively keep browning-related enzymes, specifically polyphenol oxidase and peroxidase, physically separated from their phenolic substrates located within the vacuoles (Chen et al., 2024). Because GABA prevents the leakage and mixing of these components, it successfully delays the onset of browning symptoms and maintains higher levels of total phenolics and flavonoids. Ultimately, the coordinated regulation of lipid metabolism by GABA ensures that the fruit retains its visual appeal, nutritional value, and overall marketability throughout the cold storage.

### 3.3 Algae

Algae, especially microalgae, are increasingly recognized as a sustainable source of lipids, which provides valuable components for biofuels (Kratzer and Murkovic, 2021), nutraceuticals (omega-3 fatty acids such as docosahexaenoic acid, eicosapentaenoic acid, and γ-linolenic acid), and various other bio-products (Andrew et al., 2022; Kumari et al., 2023). These algae-derived products offer promising bio-based alternatives to fossil-derived counterparts, contributing to both energy security and environmental sustainability (Wang et al., 2024). Notably, algae can accumulate lipids up to 90% of their dry weight and exhibit rapid growth, doubling their biomass within days, and enabling efficient production of value-added lipids (Pal et al., 2019). As a result, the economic utilization of microalgae is constantly increasing for bioenergy production. However, scaling up microalgae cultivation for commercialization is challenging due to rising costs and low product efficiency (Gao et al., 2023). In this regard, carbon supplementation (Tan et al., 2019) and the introduction of abiotic stress conditions, including salinity (Srivastava et al., 2017), heavy metal (Saber et al., 2024), high light intensity (Maltsev et al., 2021), nutrient starvation (Ran et al., 2019), extreme temperature (Gao et al., 2023), and nitrogen limitation (Guldhe et al., 2019), are implemented to enhance algal lipid production. While these methods have proven effective, simultaneous treatment of microalgae to stressful conditions can lead to excessive production of reactive oxygen species (ROS) (Li et al., 2021b). This, in turn, negatively impacts photosynthetic efficiency, thereby reducing the algal growth and biomass production (Shokravi et al., 2020).

To overcome the challenge of lipid accumulation and biomass growth, the use of phytohormones (Yang et al., 2022) and small chemical modulators, such as GABA, is being actively explored to perturb algal metabolism (Li et al., 2020; Zhao et al., 2020; Li et al., 2021b; Li et al., 2022; Zhao et al., 2022; Bhatnagar et al., 2024; Chakravorty et al., 2024). GABA is a non-protein amino acid found ubiquitously in bacteria (Nugraha et al., 2022), plants, animals, and microalgae. While it acts as an inhibitory neurotransmitter in mammals, in plants and microalgae, it functions primarily as a metabolite and an endogenous signaling molecule (Michaeli and Fromm, 2015). While it acts as an inhibitory neurotransmitter in mammals, in plants and microalgae, it functions primarily as a metabolite and an endogenous signaling molecule (Michaeli and Fromm, 2015). In microalgae, GABA serves as a critical component of the GABA shunt, a metabolic pathway that bypasses two steps of the TCA cycle to sustain productivity under stress (Arora et al., 2023). It is synthesized from glutamate via the enzyme GAD, a mechanism confirmed in species such as *Monoraphidium* sp. *QLY-1* (Zhao et al., 2020). This pathway becomes particularly active under unfavorable conditions, helping to maintain the cellular carbon-nitrogen balance and providing intermediates like succinate to the TCA cycle to sustain energy production and lipid biosynthesis (Li et al., 2022; Zhao et al., 2022). Under abiotic stresses such as salinity, heavy metals, or high light, microalgae rapidly accumulate intracellular GABA (Li et al., 2020; Zhao et al., 2020; Li et al., 2021b; Li et al., 2022). Acting as a signal molecule, GABA triggers a cascade of responses: it modulates cytosolic calcium levels (Ca^2+^), upregulates antioxidant enzymes (SOD, CAT, POD) to scavenge harmful ROS, and activates genes responsible for lipid synthesis (Li et al., 2020, 2022).

#### 3.3.1 Impact on lipid content and productivity

The application of GABA has consistently demonstrated a significant capacity to enhance both lipid content (percentage of dry weight) and lipid productivity (daily volumetric yield) across diverse microalgae. **Table 4** provides a comprehensive overview of these enhancements across diverse species, thereby showing GABA’s efficacy in maintaining high lipid yields even when subjected to various abiotic stresses. In *Monoraphidium* sp. QLY-1, the addition of 2.5 mM GABA increased lipid content to 55.37%, a 49.37% rise over the control while achieving a lipid productivity of 111.54 mgL^-1^d^-1^ under Cadmium (Cd^2+^) stress (Zhao et al., 2020). Similarly, when the same species was exposed to copper (Cu^2+^) stress, a combination of 1.0 mM GABA and 16 µM Cu^2+^ raised lipid content to 55.13% and productivity to 180.11 mgL^-1^d^-1^, effectively alleviating the toxicity that typically reduces biomass (Li et al., 2022). Furthermore, under conditions involving fulvic acid and salinity, a lower dose of 0.1 mM GABA was sufficient to enhance lipid content to 57.89%, which was 1.27 times higher than the control treatment (Li et al., 2020). Salinity stress has also been a key target for GABA supplementation in other species. In *Ankistrodesmus* sp. EHY exposed to 2.5 gL^-1^ NaCl, supplementation with 50 µM GABA resulted in a maximum lipid content of 59.42%. This treatment achieved a lipid productivity of 235.13 mgL^-1^d^-1^, representing a 1.27-fold increase compared to the control group (Zhao et al., 2022). In contrast, for *Pseudochlorella pringsheimii*, treatment with 2.5 mM GABA and 5 g/L NaCl increased lipid content to 29.69% (a 35.63% increase over control). Notably, this lipid gain was accompanied by a massive 93.24% increase in biomass, making the overall productivity gain significant (Bhatnagar et al., 2024).

**Table 4:** Influence of exogenous GABA supplementation on lipid production in microalgae.

Species	Concentration of GABA	Stress conditions	Outcome	Influence on lipid profile	References
*Monoraphidium* sp. *QLY-1*	2.5 mM	Cadmium (II)	Promotes the production of algal biomass and lipid synthesis, and reduces Cd accumulation by regulating C- and N- metabolism, lipogenesis gene expression, phytohormone contents and ROS signaling.	Increased levels of MUFA and reduced levels of PUFA.	Zhao et al., 2020
*Pseudochlorella pringsheimii*	2.5 mM	Salinity (NaCl)	Increase in algal biomass and chlorophyll content. Rise in protein and lipid production but decrease in total carbohydrate content.	Increases the SFA content and decreases PUFA content. But compared to the control, PUFA amount is increased.	Bhatnagar et al., 2024
*Chlorella sorokiniana*	2 mM	Chromium (III)	Promotes microalgal growth, improves biomass production and lipid accumulation in algal cells.	Boosts the production of SFA and lower the level of PUFAs.	Chakravorty et al., 2024
*Ankistrodesmus* sp. EHY	50 uM	Salinity (NaCl)	Promotes transcription of genes involved in lipogenesis, antioxidant system and cell autophagy. Also, increases the levels of intermediates in the TCA cycle and GABA shunt, resulting in lipid accumulation.	Increases the triacyl glycerol content and reduces SFA and PUFA.	Zhao et al., 2022
*Haematococcus pluvialis* LUGU	0.25 mM	Salinity + High-light	Increase in algal biomass, astaxanthin and lipid production, and decrease in ROS levels.	–	Li et al., 2021b
*Monoraphidium* sp. *QLY-1*	1.0 mM	Copper (II)	Boost in biomass and lipid production, along with increase in algal productivity and lipid content.	–	Li et al., 2022
*Monoraphidium* sp. *QLY-1*	0.1 mM	Fulvic acid + Salinity	Enhances lipid content, and biomass and lipid productivity. Improves cell growth	–	Li et al., 2020

GABA’s benefits also extend to high-value co-products and heavy metal tolerance. While *Haematococcus pluvialis* is primarily known for producing astaxanthin, 0.25 mM GABA also promoted lipid accumulation under high-light and salinity conditions. In this case, lipid content reached 55.11%, coinciding with high astaxanthin production of 3.86 mgL^-1^d^-1^ (Li et al., 2021b). Finally, in *Chlorella sorokiniana* subjected to Chromium (Cr^3+^) stress, 2.0 mM GABA stabilized growth, which is usually severely inhibited by chromium, and increased lipid content to 30%. This treatment boosted lipid productivity to 5.74 mgL^-1^d^-1^, a significant improvement over the 1.78 mgL^-1^d^-1^ observed under stress alone (Chakravorty et al., 2024).

#### 3.3.2 Impact on lipid profile

GABA not only increases the total lipid yield but also significantly influences the specific types of fatty acids produced, the lipid profile, which directly dictates the suitability of the lipids for biodiesel conversion. For instance, in *Ankistrodesmus* sp. EHY, the combination of GABA and salinity treatment shifted the profile by decreasing PUFAs while significantly increasing monounsaturated fatty acids (MUFAs), specifically C18:1 and C20:1 (Zhao et al., 2022). A similar trend was observed in *Chlorella sorokiniana*, where GABA supplementation boosted saturated fatty acids (SFAs) and simultaneously lowered PUFAs (Chakravorty et al., 2024). This shift toward higher MUFA and SFA content is highly desirable for biofuel production because it improves critical properties such as the cetane number and oxidation stability (Arias-Peñaranda et al., 2013). Consequently, the biodiesel derived from these GABA-treated cultures met international quality standards like ASTM D6751 and EN 14214 (Zhao et al., 2020; Chakravorty et al., 2024). However, this response appears to be species-specific. In contrast to the other species, GABA treatment in *Pseudochlorella pringsheimii* increased the total PUFA content compared to controls (Bhatnagar et al., 2024). While this profile may be less optimal for fuel stability, the enrichment of PUFAs is valuable for nutritional applications, such as omega-3 supplements, highlighting the versatile nature of GABA’s metabolic regulation.

#### 3.3.3 Mechanism of GABA-induced lipid metabolism

**Figure 3:**
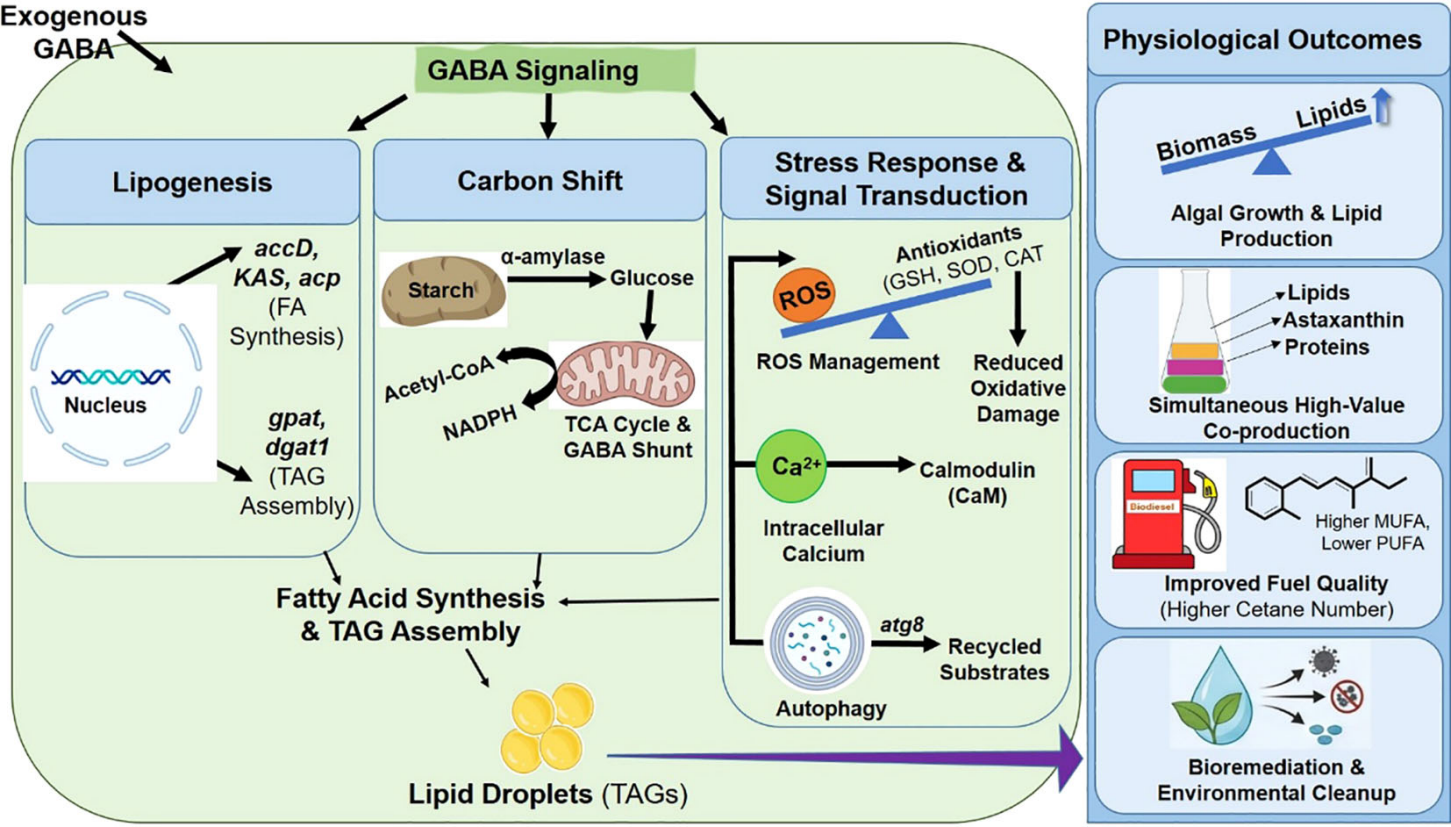
GABA-mediated lipid metabolism and its physiological outcomes in microalgae.

GABA’s primary impact on lipid metabolism involves a coordinated regulation of carbon reallocation (metabolic shift), lipogenesis gene expression, and stress-related signaling pathways, as shown in **Figure 3**.

One of its central mechanisms is the redirection of cellular carbon flow away from carbohydrate (starch) synthesis and toward lipogenesis. Under typical stress conditions, microalgae tend to store carbon as starch or carbohydrates; however, GABA signaling triggers the degradation of these reserves to provide the necessary precursors (such as acetyl-CoA) and energy for lipid synthesis. This metabolic shift is evidenced by a sharp decrease in cellular carbohydrate content which has been linked to GABA-induced upregulation of α-amylase activity (Zhao et al., 2020; Li et al., 2021b), an enzyme that promotes starch degradation. The carbon diverted from these reserves is subsequently utilized to synthesize TAGs (Zhao et al., 2020), the primary storage lipid for biodiesel production. The occurrence of this carbon shift is strongly supported by enzymatic evidence. In *Monoraphidium* sp. *QLY-1*, GABA application led to a considerable upregulation in α-amylase activity (Zhao et al., 2020). This enzymatic spike coincided directly with a reduction in starch levels and a simultaneous increase in lipid levels. Similarly, in *Pseudochlorella pringsheimii*, GABA treatment caused total carbohydrate content to decline by over 52% compared to controls while lipid content surged, confirming that the carbon backbone from starch was likely repurposed for lipogenesis (Bhatnagar et al., 2024). This metabolic shift is driven by genetic changes where GABA treatment consistently upregulates the transcription levels of key lipogenesis genes involved in the fatty acid synthesis and TAG assembly pathways. The key genes upregulated by GABA include: *accD* (acetyl-CoA carboxylase), which catalyzes the first step in fatty acid synthesis, *KAS* (3-ketoacyl-ACP synthase) and *acp* (acyl carrier protein), which are essential for fatty acid chain elongation, *gpat* (glycerol-3-phosphate acyltransferase), which initiates the assembly of fatty acids onto the glycerol backbone, and *dgat1* which catalyzes the final step in TAG formation.

The carbon units freed by starch degradation are shunted into the GABA shunt and the TCA cycle, a key metabolic pathway involved in cellular respiration. Metabolomic analyses in *Ankistrodesmus* sp. EHY confirms this redirection, showing increased levels of TCA intermediates (such as citrate, malate, and fumarate) in GABA-treated cells. This metabolic boost provides both the carbon skeletons (acetyl-CoA precursors) and the reducing equivalents (NADPH) required for fatty acid synthesis (Zhao et al., 2022). Specifically, the mitochondrial pyruvate-malate shuttle plays a crucial role where malate can be converted into pyruvic acid, offering substrates and suppressing equivalents necessary for algal lipid accumulation.

The carbon shift is not limited to recycling existing starch, as GABA also appears to enhance the uptake of new carbon to fuel lipid production. Transcriptomic analysis in *H. pluvialis* revealed that GABA treatment upregulated the expression of *rbcL*, the gene encoding the large subunit of Rubisco (Ribulose-1,5-bisphosphate carboxylase/oxygenase) (Li et al., 2021b). Rubisco is the primary enzyme responsible for carbon fixation in photosynthesis (Erb and Zarzycki, 2018). Its upregulation suggests that GABA enhances the cell’s ability to fix inorganic carbon, which is then funneled, via the carbon shift, away from protein and carbohydrate synthesis and toward the production of fatty acids and lipids.

Beyond metabolic rerouting, GABA’s influence is also deeply connected to its role in regulating cellular signaling and mitigating oxidative stress. While abiotic stress generates high levels of ROS, GABA application helps alleviate oxidative damage by reducing excess ROS to manageable levels. It achieves this by enhancing the microalgae’s antioxidant system, increasing the content of GSH, and upregulating antioxidant enzyme genes like *sod* and *cat*. For instance, in *H. pluvialis* under high-light and salinity stress, GABA upregulated *sod* and *cat* transcript levels by over 11-fold compared to controls. This significantly lowered ROS levels, protecting the cell and allowing for the simultaneous accumulation of lipids (55.11%) and astaxanthin (Li et al., 2021b). However, when complete elimination of ROS is not achieved, then GABA helps maintain a suitable level of ROS that acts as a signal transduction molecule to promote lipid synthesis. This specific level of ROS triggers the transcription of key lipogenesis genes (such as *accD*, *gpat*, and *dgat1*). This signaling role is evident in *Ankistrodesmus* sp. *EHY* under salinity stress, where GABA treatment caused a sharp, transient increase in ROS on day 1, followed by a rapid decline. This initial spike acted as a positive stress response signal to activate lipid synthesis pathways, while the subsequent decline prevented long-term damage (Zhao et al., 2022). Furthermore, lipid synthesis itself serves as a protective mechanism. ROS are often generated when there is an excess of high-energy electrons (from photosynthesis) that have nowhere to go because growth is inhibited. Fatty acid synthesis is an energy-intensive process that requires large amounts of NADPH (reducing power). By upregulating lipid synthesis, GABA creates a sink for these excess electrons, consuming the energy that would otherwise react with oxygen to form harmful ROS. This was observed in *Monoraphidium* sp. *QLY-1* under Cu^2+^ stress, where the application of GABA stimulated lipid accumulation while simultaneously reducing ROS levels. The study suggested that relieving lipid peroxidation through ROS scavenging was a prerequisite for high lipid accumulation (Li et al., 2022).

Furthermore, GABA influences other critical signaling pathways. Addition of GABA has been shown to upregulate the autophagy-related gene *atg8*, a process positively linked to the stimulation of lipid accumulation. The accumulation of GABA and the subsequent modulation of stress signals trigger the production of ethylene (ETH), another plant hormone. This GABA-induced ETH signaling specifically upregulates the expression of *atg8*, a gene marker for autophagy. The activation of autophagy allows the cell to break down damaged organelles and proteins. These recycled materials provide the raw carbon precursors and energy required to fuel the intensified lipid synthesis. The coordination is precise since autophagy, if chemically inhibited by using 3-methyladenine, the lipid-boosting effects of GABA are significantly reduced (Li et al., 2022), confirming that this recycling pathway is an essential downstream component of the GABA signaling network.

Studies have also revealed a crucial crosstalk between GABA and Ca^2+^ signaling, where both molecules mutually regulate each other to further promote the expression of lipogenesis genes. The foundation of this mechanism is the mutual regulation between GABA and intracellular Ca^2+^. Under stress conditions like salinity or heavy metal exposure, exogenous GABA application triggers an immediate increase in cytosolic Ca^2+^ levels. This calcium influx activates Calmodulin (CaM) (Li et al., 2022), a critical calcium-binding messenger protein. Crucially, this relationship is bidirectional. Elevated Ca^2+^/CaM signaling stimulates GAD activity, the enzyme responsible for synthesizing endogenous GABA. This creates a self-amplifying loop where GABA increases Ca^2+^, and Ca^2+^ increases endogenous GABA production. This amplified signal is then transmitted downstream to directly upregulate the transcription of key lipogenesis genes, such as *accD*, *gpat*, and *dgat1*, thereby initiating lipid synthesis.

#### 3.3.4 Physiological outcomes

The molecular changes driven by GABA can translate into several beneficial physiological and commercial outcomes, most notably the decoupling of stress-induced lipid accumulation from growth inhibition. GABA significantly increases lipid productivity (mgL^-1^day^-1^) by maintaining biomass production which is the primary metric driving the economic viability of algal biofuel production. Furthermore, GABA allows for the simultaneous, high-yield co-production of lipids alongside other high-value bio-products induced by stress; for example, in *H. pluvialis*, GABA facilitated the maximization of lipids, biomass, and astaxanthin (a valuable carotenoid) while enhancing protein and pigment production in other strains (Li et al., 2021b). Similarly, besides increasing the quantity of lipids, GABA also influences their quality. It alters the fatty acid profile by increasing MUFA and SFA while decreasing PUFA. This shift leads to biodiesel with a higher cetane number and greater oxidation stability, meeting quality standards. Finally, the combined GABA-stress strategy also proves effective for bioremediation where GABA application significantly reduced the intracellular bioaccumulation of heavy metals and enhanced the overall mitigation of the pollutant, demonstrating a dual function in both productivity and environmental cleanup.

## 4 Integrated summary and future directions

GABA is a conserved, multifunctional regulatory molecule that plays a pivotal role in maintaining lipid homeostasis across diverse forms of life. While traditionally recognized as a neurotransmitter, its systemic importance as a metabolic regulator is increasingly evident through its integration with the GABA shunt and the TCA cycle. In animals, GABA acts as a potent therapeutic agent against metabolic syndrome. It effectively shifts cellular metabolism from fat storage (lipogenesis) to energy expenditure (lipolysis and thermogenesis). By activating pathways such as AMPK and PKA, GABA provides a systemic defense against obesity, type 2 diabetes, and fatty liver disease. Likewise, GABA serves as a key stress regulator in plants and algae. In plants, it preserves the integrity of cell membranes during environmental stress, such as cold, by preventing phospholipid degradation, while in microalgae, it facilitates a metabolic shift, redirecting carbon from starch reserves to lipid synthesis, thereby enhancing both the quantity and quality of lipids for sustainable biofuel production. Ultimately, the GABA-lipid relationship represents a critical intersection of carbon and nitrogen metabolism that is fundamental to metabolic health and environmental resilience.

Looking forward, although the biochemical pathways involved in GABA-mediated lipid metabolism are becoming clearer, several research gaps must be bridged to fully harness this molecule for therapeutic and commercial applications. A primary hurdle is the transition from rodent models to rigorous, longitudinal human clinical trials to establish the optimal dosage, safety, and efficacy of GABA-rich functional foods in treating chronic metabolic disorders. Future investigations should also delve into the gut-brain axis, specifically exploring how GABA-producing gut microbiota influence host lipid profiles which could pave the way for innovative postbiotic treatments for obesity. Furthermore, the precise molecular crosstalk between GABA and other signaling mediators, such as calcium ions, ROS, and phytohormones like ethylene and abscisic acid, requires further elucidation. To achieve this, the integration of advanced multi-omics technologies (transcriptomics, proteomics, and lipidomics) will be essential to map how these complex signals are fine-tuned across different species.

In agriculture, expanding the use of exogenous GABA to improve the shelf-life and stress-tolerance of a wider variety of commercial crops offers a tangible strategy to reduce postharvest losses and strengthen global food security. In the energy sector, optimizing the GABA-induced metabolic shift in microalgae through high-throughput bioreactor technologies could revolutionize the scalability of sustainable biofuel production. In summary, leveraging the GABA-lipid connection offers a dual opportunity, advancing precision therapy for metabolic health and developing sustainable biotechnological solutions for the global energy and food crises.

## 5 Conclusions

The regulation of lipid metabolism by GABA is a fundamental biological mechanism conserved across diverse biological forms of life. This review has highlighted that GABA functions as more than a signaling molecule. It acts as a metabolic regulator, diverting carbon flux through the GABA shunt while simultaneously modulating the profile of key lipogenic and lipolytic genes. By regulating the expression of factors such as SREBPs, PPARs, and fatty acid desaturases, GABA coordinates shifts in lipid composition and storage. In animals, this axis offers a promising therapeutic target for metabolic disorders through upregulation of lipolytic and thermogenic pathways, enhanced fatty oxidation, and activation of PKA signaling pathways. In plants and algae, the GABA-lipid connection, driven by the up-regulation of stress-responsive genes, is a critical component of the stress response, facilitating membrane integrity and energy storage under environmental flux. Hence, the metabolic crosstalk between GABA and lipid metabolism can serve as the cornerstone for maintaining health and sustainable biotechnology.
